# Obliteration of oral and maxillofacial region by SARS-CoV-2 infection

**DOI:** 10.3205/dgkh000558

**Published:** 2025-06-16

**Authors:** Karthik Shunmugavelu, Jayakanthan Saravanan

**Affiliations:** 1 Department of Dentistry, Oral and Maxillofacial Pathology, PSP medical college hospital and research institute Tambaram Kanchipuram, India; 2 Sree Balaji Medical College and Hospital, Chrompet, Chennai, Tamil Nadu, India

**Keywords:** COVID-19, oral, facial, dental, pathology

## Abstract

COVID-19 present as fever, cough, dyspnea, and pneumonia. The infection causes orofacial manifestations in several cases but seems to be underreported, mainly due to the lack of oral examination of patients with suspected and/or confirmed COVID-19. Described are COVID-19- related oral lesions that can be the first and/or the unique manifestation of the disease.

## Abstract (de)

COVID-19 ist charakterisiert durch Fieber, Husten, Dyspnoe und Pneumonie. In manchen Fällen manifestiert sich die Infektion orofazial, worüber anscheinend weniger häufig berichtet wird, hauptsächlich aufgrund der fehlenden oralen Untersuchung von Patienten mit Verdacht auf und/oder bestätigter COVID-19 Erkrankung. Beschrieben werden COVID-19-bedingte orale Läsionen, die die erste und/oder einzige Manifestation der Krankheit sein können.

## Introduction

The contribution of the oral cavity to COVID-19 pathogenesis and transmission has been little explored. It is unknown whether SARS-CoV-2 can infect and replicate in the oral mucosa or glands [1], [2]. This is critical because if the glands or mucosa are sites of early infection, they may play an important and underappreciated role in transmitting virus “intermucosally” to the lungs or gastrointestinal tract. Alternatively, saliva may also play a central role in transmitting the virus extraorally in asymptomatic, pre-symptomatic, or symptomatic individuals [3], [4].

The human oral cavity is a diverse collection of tissue niches with potentially unique vulnerabilities to viral infection. These sites include oral mucosae (hard palate, buccal mucosa, dorsal and ventral tongue) as well as the terminally differentiated secretory epithelia of the minor saliva glands (distributed in the buccal and labial mucosa, hard and soft palate, ventral and dorsal tongue) and major saliva glands (parotid, submandibular, and sublingual) [5], [6]. Nearby are diverse oropharyngeal niches (palatine and lingual tonsils, soft palate). Saliva, a mixture of fluids, electrolytes, proteins, and cells (immune and sloughed mucosal epithelial cells) is made primarily by the saliva glands and empties into the oral cavity where it mixes with other fluids (crevicular fluid) and cells.

SARS-CoV-2 uses host entry factors, such as ACE2 and TMPRSS family members (TMPRSS2 and TMPRSS4). ACE2 and TMPRSS2 expression has been reported in oral tissues. Expression matrices, including a low-frequency ACE2/TMPRSS2 co-expressing cells in basal 1, ducts, mucous acini and myoepithelial clusters [7], [8].

## Oral manifestations of COVID-19

### Ulcer and erosions

One of the most common oral complications associated with COVID-19 confirmed or suspected individuals are ulcerative lesions of the oral cavity. The site, pattern and presentation vary. Tongue (dorsum and lateral boarder) is the most common reported site followed by hard palate and buccal mucosa. Irregular and painful ulcers either appear alone (single ulcers) or in the form of multiple tiny ulcers. Clusters of ulcers either resemble herpetiform ulcers or multiple apthoid ulcers with diffuse erythematous base. These multiple aphthoid ulcers later on coalesce to form large ulcers with yellowish fibrin covering them, resembling erythema multiform-like disease. Ulcers, erosions and blood crust on labial mucosa along with palatal and gingival petechiae are also reported [9], [10].

### Vesiculobullous lesions

These lesions mostly appear in association with cutaneous manifestations and show a range of presentation such as blisters, petechiae, erythematous lesions and erythema multiform-like lesions. Tongue and palate (soft and hard) are the most common reported location of these lesions. Erythema multiform-like lesions are most commonly reported lesions accompanied by skin target lesions [11], [12].

### Plaques

Candidal plaque-like lesions are also observed in association with COVID-19 both red; and white plaques. They are located on the dorsum of the tongue and palate. They were also observed along with multiple tiny ulcers, taste changes, tongue and masticatory muscles pain. Immune system suppression because of antibiotic therapy, deteriorating general health and neglected oral hygiene can be possible causes of these plaques [13], [14].

### Reactivation of HSV

Hedou et al. [15] while reporting cutaneous manifestations of COVID-19 in 103 patients found reactivation of herpes simplex in one intubated patient in intensive care. Although cutaneous manifestations disappeared with median time of 48 h (from 24 h to 6 days), no information regarding resolution of Herpes simplex was provided. Studies have also reported multiple tiny yellow ulcers on dorsum of the tongue that resembled to late stage of herpetic recurrent infection along with geographical tongue [16], [17].

### Angina bullosa

These blood filled blisters are observed on soft palate, tongue and cheek. They are brown– black single or multiple lesions and may appear after initiation of therapies for COVID-19 [18].

### Gingival changes 

Gingival changes such as generalized erythematous and edematous gingivae, gingivo-parodontal bleeding, necrotic interdental papillae and desquamative gingivitis are reported in the literature in critically ill patients with neglected oral hygiene. In a COVID-19 suspected patient symptoms disappeared within 10 days of antibiotics and topical antiseptic mouthwash usage [19], [20].

### Dry mouth

Dry mouth is also reported in association with COVID-19 positive patients. In a study, 16 patients reported dry mouth along with other symptoms (PCR positive for COVID-19). This number reduced to 1 when PCR for the disease became negative. In another cross-sectional study, 72 patients with COVID-19 reported dry mouth [21].

### Gustatory changes

Gustatory and olfactory changes can be the only symptom in mild cases of COVID-19 or the initial symptom in patients who ultimately present with more severe respiratory failure. The reported gustatory changes associated with COVID-19 are hypogeusia, dysgeusia and ageusia [22]. It is reported that angiotensin-converting enzyme 2 (ACE2) cell receptors are expressed in abundance on respiratory epithelium and oral mucosa especially tongue. SARS-CoV-2 has a great affinity for these receptors. Direct damage to nasal and oral epithelium and neuroinvasive nature of this virus can result in olfactory and gustatory disorders [23]. Symptoms such as halitosis, tongue and masticatory muscle pain and swelling, geographical tongue, hyperplasia of papilla associated with taste changes and macroglossia have also been reported along with fatigue and major symptoms of COVID-19 in few case reports [24], [25], [26], [27].

## Conclusion

COVID-19 patients may present with ulcerative, erosive, vesicobullous and plaque-like oral lesions. Further research is needed to confirm a link between reported mucosal lesions and COVID-19, as these lesions may be the first sign of the disease or secondary to medications, reduced immunity, vascular compromise, localized or generalized inflammation and neglected oral hygiene. Dental professionals should be aware of oral manifestations, predisposing factors and underlying mechanisms while examining and before initiating any treatment in patients. 

## Notes

### Competing interests

The authors declare that they have no competing interests.

### Funding 

None.

### Authors’ ORCIDs

- Karthik Shunmugavelu: 0000-0001-7562-8802
- Jayakanthan Saravanan: 0000-0002-4965-8829
